# Interactions between Indigenous Endophyte *Bacillus subtilis* L1-21 and Nutrients inside Citrus in Reducing Huanglongbing Pathogen *Candidatus* Liberibacter Asiaticus

**DOI:** 10.3390/pathogens10101304

**Published:** 2021-10-12

**Authors:** Suhail Asad, Pengbo He, Pengfei He, Yongmei Li, Yixin Wu, Ayesha Ahmed, Yunyue Wang, Shahzad Munir, Yueqiu He

**Affiliations:** 1 State Key Laboratory for Conservation and Utilization of Bio-Resources in Yunnan, Yunnan Agricultural University, Kunming 650201, China; sohailasad74@gmail.com (S.A.); pengbohe@126.com (P.H.); nanhudaozhu@163.com (P.H.); kala.111@163.com (Y.L.); WY68579@126.com (Y.W.); aisha_ahmed01@hotmail.com (A.A.); yunyuewang@126.com (Y.W.); 2 Faculty of Agronomy and Biotechnology, Yunnan Agricultural University, Kunming 650201, China

**Keywords:** *Candidatus* Liberibacter asiaticus, endophyte, citrus Huanglongbing, nutrients, *Bacillus subtilis* L1-21

## Abstract

Huanglongbing (HLB) pathogen *Candidatus* Liberibacter asiaticus (*C*Las) brings a great concern about the phloem nutrient transport in diseased plants. There is an urgent need to find the best management strategies to reduce the losses in the citrus industry worldwide. Endophytic bacteria are negatively affected by *C*Las pathogen, and these endophytes are associated with improved availability of nutrients and pathogen resistance. This study underpins the relationship between *C*Las pathogen, endophyte population and nutrients availability in citrus plants. The citrus plants were treated with *Bacillus subtilis* L1-21 and Hoagland solution to find out synergism efficacy to mitigate citrus HLB. We showed that citrus shoots in the presence of 50% Hoagland solution displayed maximum number of endophytes with 6.28 × 10^3^ to 3.04 × 10^5^ CFU/g. Among 50 candidate strains, *B. subtilis* L1-21 emerged as potential antagonist against surrogate strain *Xanthomonas citri* subsp. *citri*. The citrus half-leaf method identified that application of endophyte L1-21 with 50% Hoagland solution successfully reduces the *C*Las abundance. We point out that this combination results in a higher number of endophytes population with 2.52 × 10^4^ to 9.11 × 10^6^ CFU/g after 60 days, and reduces *C*Las pathogen abundance in asymptomatic HLB plants. In HLB symptomatic citrus plants, *B. subtilis* L1-21 potentially increases the endophyte population from 1.11 × 10^4^ to 5.26 × 10^7^ CFU/g in the presence of Hoagland solution, and pathogen abundance was reduced from 9.51 × 10^5^ to 1.06 × 10^4^ copies/g. Altogether, we suggested that the presence of endophyte L1-21 with Hoagland solution is more effective in HLB asymptomatic citrus plants, but a slight reduction of pathogen was observed in symptomatic plants. The findings revealed the role of indigenous citrus endophyte *B. subtilis* L1-21 along with other nutrients in the reduction of *C*Las pathogen abundance inside symptomatic and asymptomatic plants in citrus endophyte–nutrient–pathogen interplay.

## 1. Introduction

Citrus greening disease, commonly known as citrus Huanglongbing (HLB) is one of the most devastating citrus diseases causes extreme economic losses globally [1]. The disease-causing pathogens are non-culturable, fastidious, phloem-restricted α-proteobacteria named as *Candidatus* Liberibacter asiaticus (*C*Las), *Candidatus* Liberibacter americanus (*C*Lam), and *Candidatus* Liberibacter africanus (*C*Laf) based on their origin [1,2,3]. The vector responsible for transmission of pathogens is Asian citrus psyllid, known as *Diaphorina citri* [4]. In diseased plants, pathogen moves into the phloem of the citrus leaves and causes sieve plug formation and accumulation. Subsequently, this accumulation alters the metabolism of carbohydrates and blocks nutrient transport result in phenotypic changes to the citrus tree in the form of reduced root growth, blotchy, and yellow mottles on the leaves [5,6]. The distribution of HLB pathogen is highly patchy, as most of the bacterial titers can be found in the leaf midribs, diseased roots and stems [7,8].

Of interest is the difficulty in HLB symptoms diagnosis [9], specially, leaf symptoms of HLB infected citrus plants could be confused with that of nutrient deficiency at early stage of infection [10,11,12]. At initial stage of HLB infection, leaf symptoms resemble with Zn, Fe, and Mg deficiencies [7,13]. Later severe symptoms as enlarged, swollen, and corky leaf veins resemble B deficiency [12]. Moreover, some of those symptoms are related to starch accumulation in leaves [9], resembling with Ca, Mg and Zn deficiencies [14,15]. HLB-affected sweet orange leaves (*Citrus sinensis*) reported lower amount of K, Ca, Mg, Cu, Fe, Zn, Mn, and B as compare to healthy leaves [6,15,16].

Endophytes live asymptomatically inside plant building the microbiome and share most intimate association with their host. They are even regarded as extended genomes of plant for the virtues they perform for plant health and safety. Employment of indigenous endophytes isolated from healthy state to engineer the diseased citrus endophytic microbiomes that can provide sustainable solution for vascular pathogens [17,18].

To date, endophyte-mediated resistance always emerge as an exciting possibility to manage different diseases in the labs and field experiments [19]. One long standing proposed role of endophytic bacteria in plant disease control is to assist the plant to uptake nutrients, improve stress tolerance and provide pathogen resistance [20,21,22]. Pathogen resistance activities are associated with secondary metabolites production, antibiotics or chitinase enzyme that can inhibit the growth of plant pathogens. All of these studies support the idea of using indigenous endophytes as potential antagonist. Their effective use will reduce the dependence on chemical fertilizers and pesticides [23]. Keeping the importance of previous studies in citrus plants using endophyte L1-21, remarkable changes were observed in healthy and diseased citrus plants [24]. Metabolomics approaches were conducted to gain information about the molecular mechanism of citrus plant to defend themselves against *C*Las [25]. This strain inoculation to the postharvest gray mold of tomato considerably reduces the pathogen *Botrytis cinerea* [25,26]. Therefore, the main goal of this study was to assess disease-nutrient model for comparing HLB symptoms with nutrient deficiency in the presence of endophyte *B. subtilis* L1-21, to study the relationship between symptoms of citrus HLB and nutritional deficiency and to examine the role of endophytes to reduce the *C*Las pathogen abundance and nutritional availability.

## 2. Results

### 2.1. Effect of Hoagland Solution on Citrus Endophytic Population in Citrus Shoots

**Figure 1:**
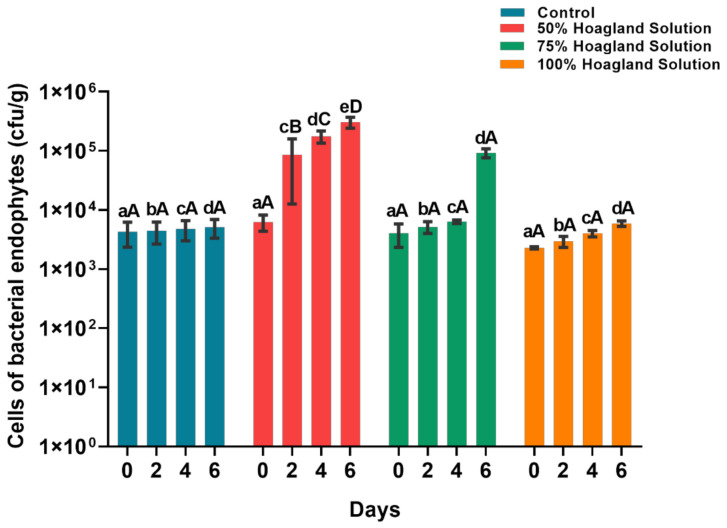
Population dynamics of endophytes in citrus shoots before and after application of different concentrations of Hoagland solution (50%, 75%, 100%) and control (ddH_2_O). Data were analyzed using analysis of variance (ANOVA) followed by Duncan’s multiple range test (*p* < 0.05). Different letters on top indicate significant differences between different treatment and error bars indicate the standard error of the mean (SEM). Small letters (abc) denote the difference between groups at same day, while capital letters (A,B,C) indicate the difference within same group at different days. Each treatment consists of three replicates and each replicates consist of 6 citrus shoots.

The effect of different concentrations (50%, 75% and 100%) of Hoagland solution on endophytes population in citrus shoots indicated that treatment of 50% Hoagland solution significantly increased the number of endophytes in citrus shoots (6.28 × 10^3^ to 3.04 × 10^5^ CFU/g) followed by 75% (4.06 × 10^3^ to 9.22 × 10^3^ CFU/g). The increase concentration of tested solution (100%) did not change the endophyte population inside citrus shoots and it was found to be stable at 10^3^ CFU/g of citrus leaves. No difference of endophyte population (10^3^ CFU/g in all three) was found among the two concentration of Hoagland solution (75 and 100%) and the control (Figure 1).

### 2.2. Antagonistic Effect of Different Bacillus *sp.* against Xanthomonas citri *subsp.* citri

The 50 candidate strains isolated in our study were tested for suppression efficiency against surrogate strain *Xanthomonas citri* subsp. *citri*. We suggested that among all of these tested endophytic strains, six strains (*Bacillus subtilis* YN-003, *B. subtilis* YN-012, *B. spizizenii* YN-016, *B. subtilis* YN-024, and *B. subtilis* YN-033 and *B. subtilis* L1-21) displayed significant antagonistic activities against *X. citri* subsp. *citri.* The results indicated that endophyte *B. subtilis* L1-21 was found to have maximum inhibition zone between other treatments. The growth inhibition ratio (GIR) of *B. subtilis* L1-21 (0.63%) was higher than the other *Bacillus* species (Table 1).

### 2.3. Effect of Bacillus *sp.* and Hoagland Solution on CLas Abundance by Using Half-Leaf Method

To further associate the use of endophyte and nutrients solution, the citrus half-leaf method enabled our understanding about pathogen reduction in one half of leaf, while the other half was kept as control. The experiment revealed that endophytic bacteria *B. subtilis* YN-003, *B, subtilis* YN-012, *B. spizizenii* YN-016, *B. subtilis* YN-033 and *B. subtilis* L1-21 with combination of Hoagland solution significantly reduce the *C*Las abundance from initial pathogen abundance of 1.47 × 10^6^, 5.96 × 10^5^, 6.21 × 10^4^, 9.29 × 10^6^, 1.65 × 10^5^ to 9.11 × 10^5^, 4.48 × 10^5^, 4.16 × 10^3^, 1.17 × 10^6^, 7.52 × 10^3^, respectively (Table 2). Maximum pathogen reduction was recorded after 4 days in the endophyte *B. subtilis* L1-21 combination with Hoagland solution highlights the considerable reduction of *C*Las pathogen (Table 2).

### 2.4. Bacillus Subtilis L1-21 Successfully Reduce the Pathogen Inside Asymptomatic Citrus Plants in the Presence of Hoagland Solution

**Figure 2:**
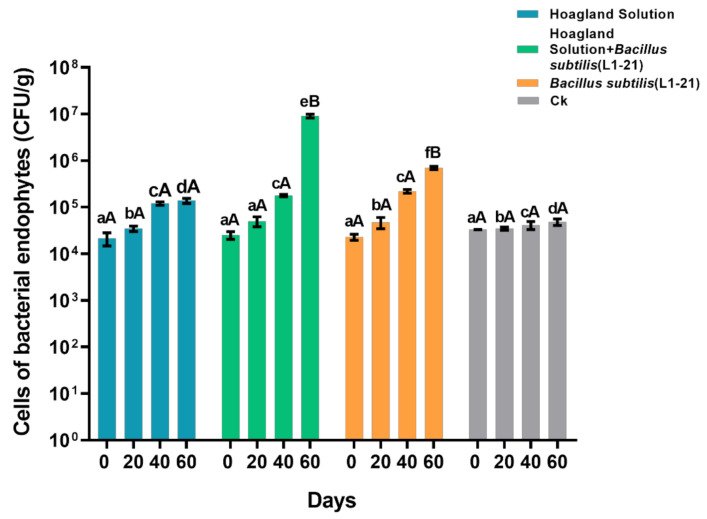
Population dynamics of endophytes in HLB asymptomatic citrus plants before and after application of different treatments (50% Hoagland solution, 50% Hoagland solution+ *B. subtilis* L1-21, *B. subtilis* L1-21 and control (ddH_2_O)). Data were analyzed using analysis of variance (ANOVA) followed by Duncan’s multiple range test (*p* < 0.05). Different letters on top indicate significant differences between different treatment and error bars indicate the standard error of the mean (SEM). Small letters (abc) denote the difference between groups at same day, while capital letters (A,B,C) indicate the difference within same group at different days.

Foliar application of different treatments (50% Hoagland solution, 50% Hoagland solution + *B. subtilis* L1-21, *B. subtilis* L1-21) on asymptomatic *Cirtus limon* plants grown in greenhouse showed considerable pathogen reduction. We showed maximum number of endophyte population varies from 2.52 × 10^4^ to 9.11 × 10^6^ CFU/g when the citrus plants were treated with endophyte L1-21 and nutrient Hoagland solution. The application of Hoagland solution and *B. subtilis* alone results in endophyte dynamic of 2.15 × 10^4^ to 1.38 × 10^5^ and 2.30 × 10^4^ to 7.01 × 10^5^ CFU/g, respectively) (Figure 2). Although, the number of endophytes increased considerably in asymptomatic plants by application of different treatments. However, it’s also necessary to know the impact of endophyte population in reduction of pathogen abundance. Meanwhile, leaf samples were collected to check pathogen abundance through real time qPCR analysis with a 20 days interval. After 60 days, results indicated that citrus plants treated with 50% Hoagland solution+ *B. subtilis* L1-21 reduced the pathogen abundance up to 91.43% from 3.20 × 10^2^ to 2.74 × 10^1^ compared with the treatment of Hoagland solution and endophyte L1-21 alone range from (5.46 × 10^2^ to 1.69 × 10^2^, 5.13 × 10^2^ to 4.12 × 10^2^, and 2.95 × 10^2^ to 9.57 × 10^2^ CFU/g, respectively) (Table 3).

### 2.5. Slight Pathogen Reduction in Symptomatic Citrus Plants in the Presence of Hoagland Solution + Bacillus subtilis L1-21

**Figure 3:**
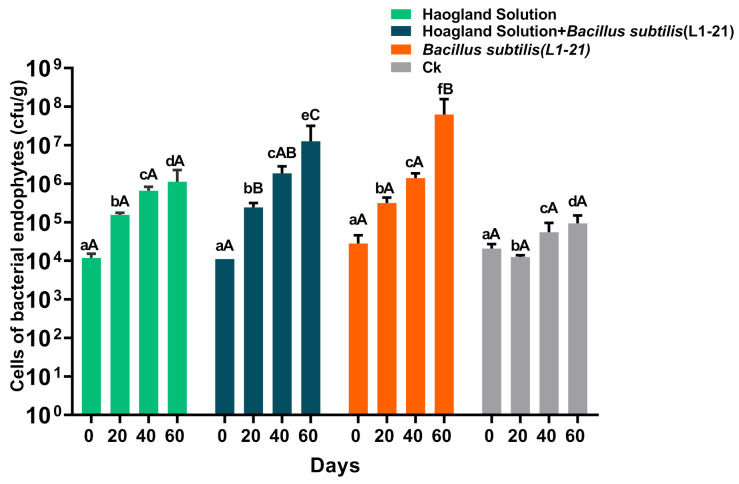
Population dynamics of endophytes in HLB symptomatic citrus plants before and after application of different treatments (50% Hoagland solution, 50% Hoagland solution+ *B. subtilis* L1-21, *B. subtilis* L1-21 and control (ddH_2_O)). Data were analyzed using analysis of variance (ANOVA) followed by Duncan’s multiple range test (*p* < 0.05). Different letters on top indicate significant differences between different treatment and error bars indicate the standard error of the mean (SEM). Small letters (abc) denote the difference between groups at same day, while capital letters (A,B,C) indicate the difference within same group at different days.

Besides treatment in asymptomatic citrus plants, same applications were also tested in symptomatic *C. limon* plants. Similar trends of maximum endophyte population were found in the plants treated with 50% Hoagland solution+ *B. subtilis* L1-21 range from 1.11 × 10^4^ to 5.26 × 10^7^ CFU/g. In contrast, treatment with *B. subtilis* L1-21, and 50% Hoagland solution represents the endophyte population as 3.81 × 10^4^ to 6.26 × 10^7^, and 1.19 × 10^4^ to 1.12 × 10^6^ CFU/g, respectively (Figure 3). The endophyte increase led to pathogen reduction in diseased citrus plants, as we highlighted that endophyte L1-21 and Hoagland solution combination reduces that pathogen abundance to 98.88% after 60 days, which are not significantly different from the endophyte L1-21 treatment alone (97.45%). The Hoagland solution applied independently slightly reduce the *C*Las pathogen with 41.38% (Table 4). HLB-affected citrus plant treated with *B. subtilis* L1-21 have changed leaf color and improved the growth with reduction of *C*Las titer (Data not shown). The experiment results concluded that *B. subtilis* L1-21 have possible effect on Hoagland solution with stable concentration in order to increase the endophyte population and reduce the *C*Las pathogen, but how the endophyte interaction is involved in the present of these nutrient solution need to study in future work.

## 3. Discussion

Endophyte-mediated control of citrus HLB is a serious challenge for saving the citrus industry globally [27]. Disruption of vascular function, loss of root mass, and altered mineral nutrition in HLB-affected trees lead to arrested tree and fruit growth, increased fruit drop, a decline in production, and could eventually lead to tree death [28,29]. Currently, HLB causes 90% production loss in commercial citrus orchards [30]. During past several years, many citrus growers have been documented to use nutrient management strategies for enhancing citrus production in HLB- affected trees [31], while the role of endophytic bacteria in plant disease control is to assist the plant to uptake nutrients, improve stress tolerance and provide disease resistance [20,21].

As previous studies proved that *B. subtilis* isolated from healthy chestnut trees have shown strong antagonistic activity against *Cryphonectria parasitica*, a causal organism of chestnut blight [32]. Different endophytic bacteria *B. amyloliquefaciens*, *B. subtilis* and *B. pumilus* reported as producers of several antibiotics (surfactin, iturin, bacillomucine; azalomycin F, surfactin, arthrobactin; surfactin, amphomycin, arthrobactin and valinomycin) which are highly inhibitory to the growth of *X. campestris* pv. *campestris* [33]. *Bacillus subtilis* was reported as a biocontrol agent against bacterial blight of rice through seedling dip, soil and foliar application [34]. *Bacillus subtilis* QST 713 is a commercialized bacterial strain used in biocontrol programs around the world [35]. As reported previously, there is always some high competition among nutrients in the plant that can negatively affect each other [36,37]. The citrus plant which was highly infected by HLB, has recovered after 60 days of foliar application of *B. subtilis* L1-21. Our main focus in this study was to check whether nutrients application could have any reduction of pathogen or increase population of endophytes. We showed the number of endophytes were constant applying different concentrations. Low and high trends of endophytes were present indicating that nutrients may have any direct role in activating the role of other endophytes that can reduce the pathogen inside, but how the interaction takes place still needs to be verified in detail through molecular mechanisms.

Based on overall literature, the present study was designed to check the Hoagland solution and endophyte dynamics in citrus plants during inoculation of isolated endophyte *B. subtilis* L1-21. In our knowledge, it is the first time Hoagland solution was applied on citrus plants with combination of endophyte *B. subtilis* L1-21 to find relationship of nutritional application and biocontrol strategy for reduction of *C*Las titer. The Hoagland solution provides every essential nutrient required by green plants and is appropriate for supporting growth of a large variety of plant species [38]. It consists of high concentrations of N and K, which make it useful as a hydroponic nutrient solution for the development of large plants like tomato and bell pepper [39]. Firstly, we have applied Hoagland solution for citrus shoots by hydroponic method to find out the role of nutrients in endophytes colonization. Initial findings of the study showed that Hoagland solution with 50% concentration has shown good compatibility with colonization of indigenous endophytes inside leaves of citrus shoots. We suggested increase endophyte dynamics inside citrus plants, and higher level could not show any positive or negative effect on native endophytes.

Macro-and micro-nutrients can further improve plant disease resistance against *C*Las pathogen indirectly through modification of microbial communities [40]. Plant microbiomes are the key agents involved in plant disease resistance [41]. Thereby, enhanced micronutrient fertilization could be used to improve plant growth in the presence of pathogen. In addition, micronutrients can trigger a systemic acquired resistance in plants, so they would work as elicitors to reduce disease loses, and also inhibit the pathogen colonization [42]. Specific function of different micronutrients in reduction of *C*Las titer specially, Zn can reduce bacterial infection. Mn is a key component for non-structural carbohydrate formation, for N metabolism, and for phenols and phytoalexin production, and finally, Cu has been used since the nineteenth century to reduce phytosanitary issues caused by microorganisms [43]. Taking together, we suggested that increasing micronutrient concentrations in diseased plants through both foliar and soil application could reduce HLB loses in citrus trees.

As previous studies, endophyte *B. thuringiensis* and *B. subtilis* (S-12) was reported as biocontrol of citrus canker caused by *X. axanopodis* pv. *citri* [44]. Same as, we also proved that *B. subtilis* L1-21 have antagonistic activity against *X. citri* subsp. *citri.* Endophytes with and without combination of Hoagland solution were further applied on HLB-affected citrus leaves by half-leaf method. Our study proved that *B. subtilis* L1-21 with combined treatment of Hoagland solution have shown maximum reduction of *C*Las titer. This endophytic bacterial strain along with the nutrient can possibly manage pathogen within weeks, but more time is required to get more results, as this pathogen is slow growing inside citrus plants, making the research difficult to handle inside citrus plants.

Furthermore, in order to get a clearer picture of using these endophytes, *B. subtilis* L1-21 with combined application of Hoagland solution was applied on HLB asymptomatic and symptomatic citrus plants. We concluded that the number of pathogen abundance reduced with an increasing number of endophytes. Maximum disease reduction was observed in HLB symptomatic plants by treatment of *B. subtilis* L1-21, and combined treatment of Hoagland solution with *B. subtilis* L1-21 in HLB asymptomatic plants displayed remarkable changes in *C*Las titer. Overall, comparison of Hoagland with endophyte application in asymptomatic and symptomatic citrus plants revealed clear difference, with maximum number of pathogen reduction was noted in former and slight pathogen reduction in later plants. We suggested that Hoagland solution could helpful in asymptomatic plants by providing nutrients and improving growth structure.

Importantly, restructuring the diseased host in the presence of inoculated endophytes with nutrients can displayed marked changes in citrus host, as our results revealed that endophyte application along with nutrients results in reduction of pathogen and same time the number of endophytes also reached to remarkable level. Often researchers do not see improvement in growth or yield of HLB-affected trees in only 1 to 2 years of HLB-nutrition research [45]. This suggests that HLB-nutrition research, like healthy citrus research, should be conducted for longer durations to appropriately evaluate the treatments, particularly with large, mature trees. Still, a lot of research has been used to mimic the spread of HLB disease in citrus groves, and there are still doubts about the contribution of such strategies to minimize the losses caused by the disease in the citrus industry.

Zambon et al. [46] reported beneficial effects of Mn foliar application with three times higher than the recommended dose in HLB-affected trees have shown reduction of *C*Las titer and improvement of fruit production [46,47]. Therefore, it can be proven that nutrients have great importance in the interaction of the plant host, microbial community and vectors [14,46,48]. Most of essential nutrients influenced the severity of plant disease [49]. It seems that nutrient elements could reduce the severity of disease symptoms, yet there are many findings on both sides of this debate [50].

Nutrient application via foliar sprays or soil drenching reported as another tool to protect HLB-affected trees [51]. Soil fertility and observing exact plant nutritional status is a key strategy to fulfill the plant desire. It is very important for citrus grower to have grip on knowledge of proper dosses of fertilizer application and timing for better plant growth. By enhanced nutritional programs, citrus HLB-affected trees can also survive and increase fruit production [11]. There is an interesting approach for better fruit production in the presence of disease by proper irrigation, best cultural practices and maintaining nutritional requirements through foliar and soil drenching techniques [52].

## 4. Materials and Methods

### 4.1. Bacterial Strains, Culture Conditions, and Citrus Plants

The candidate bacterial endophytes *Bacillus subtilis* YN-003, *B. subtilis* YN-012, *B. spizizenii* YN-016, *B. subtilis* YN-024, and *B. subtilis* YN-033 were isolated from healthy citrus plants (*Citrus limon*, *Citrus sinensis*) and cultured on Luria Bertani (LB; Bacto tryptone 10 g/L, yeast extract 5 g/L, NaCl 5 g/L, agar 18 g/L, and pH 7.0) and Tryptic Soya Agar (TSA) media. *Bacillus subtilis* L1-21 was previously isolated as a potential indigenous endophyte from healthy citrus plants [25]. Healthy citrus shoots (*Citrus Limon*) were collected from citrus plants grown in pots in a greenhouse of State Key Laboratory for Conservation and Utilization of Bio-resources Kunming, China.

### 4.2. Effect of Hoagland Solution on Endophytes Population in Citrus Shoots

Hoagland solution was prepared according to the designed formula of Hoagland and Arnon in 1938 which was revised by Arnon in 1950 [53]. Indigenous endophytes were isolated from citrus leaf through surface sterilization with 75% ethanol for 30 s, followed by NaClO for 1 min. Leaves were ground in a sterilized pestle and mortar, followed by three series of dilutions (10^−1^, 10^−2^ or 10^−3^). Furthermore, 100 µL of suspension was taken from 10^−3^ dilution and spread on LB agar and incubated at 30 °C for 48 h [51,52]. Endophyte colonies were counted and identified on morphological basis. Different concentrations of Hoagland solution (50%, 75% and 100%) were treated to check the efficacy for maximum number of endophytes colonization. Citrus shoots treated with ddH_2_O serve as control. Initially, leaf samples were collected before application, followed by 2 days interval with the presence of Hoagland solution to check endophyte population dynamics inside citrus leaves.

### 4.3. Antagonistic Effect of Candidate Bacillus strains against Xanthomonas citri *subsp*. citri

Different endophytes (50 strains) were isolated and identified on the basis of morphological characters. These endophytes were further cultured on TSA (Tryptic soy agar) liquid media and incubated in shaker at 30 °C for 16 h, all of these isolates were tested on TSA media plates for confirmation of biocontrol activity. Due to non-culturable characteristics of pathogen *Candidatus* Liberibactor asiaticus, surrogate pathogen was used to test candidate endophytes in lab experiments. *Xanthomonas citri* subsp. *citri* was used as pathogenic bacteria with dilution of 10^−1^ and concentration of 200 µL was spread on TSA agar media plates. After 15 min of inoculating *X. citri* subsp. *citri*, 5 µL culture of each isolated strain were punched on the plate by pipetting followed by incubation for 48 h at 28 °C. The experiment was repeated four times. Diameter of bacterial inhibition zones was measured and suppression efficiency of isolates were evaluated. In addition, the bacterial isolates with maximum inhibition zones were tested again against pathogen. Antagonistic activity was expressed as the growth inhibition ratio (GIR) by using the following formula.

GIR = [(Inhibition zone diameter − colony diameter)/inhibition zone] × 100(1)

### 4.4. Effect of Different Bacillus *sp.* on Number of Pathogen Abundance of CLas Using Half-Leaf Method

Diseased leaves were selected from HLB-affected citrus plants grown under greenhouse of Yunnan Agricultural University, China and confirmed through qPCR using primer sequence CQULA04F 5′ TGGAGGTGTAAAAGTTGCCAAA 3′, CQULA04R 5′ CCAACGAAAAGATCAGATATTCCTCTA 3′. Half leaf methods were applied as designed by Munir et al. [24], with some modifications. Leaves were further treated with different candidate endophytes. Leaves were cut into two parts; a half part was firstly analyzed for checking number of pathogen abundance through qPCR before treatment of endophytes. Different *Bacillus* strains (*B. subtilis* YN-003, *B. subtilis* YN-012, *B. spizizenii* YN-016, *B. subtilis* YN-033 and *B. subtilis* L1-21) with and without Hoagland solution and Ck (ddH_2_O) were treated on half parts of leaves to check efficacy of endophytes and Hoagland solution in reduction of *C*Las titer. Hoagland solution was prepared as described above. For DNA extraction, midribs of leaves were cut and frozen in liquid nitrogen, followed by midribs of 5 leaves from one tree were pooled and macerated in a sterile pestle and mortar. DNA of leaf samples was extracted using the method described by Munir et al. [27] with slight modifications. The *C*Las pathogen was detected using SYBER Green reagent, and the PCR reaction was performed in a 20 µL reaction mixture containing 1xPCR buffer (SYBER Green Master Mix; Bio-Red, Hercules, CA, USA); 0.8 µL of CQULA04R and CQULA04F primer and DNA template required for reaction. Detection of HLB pathogen through qPCR was checked by followed method described with slight modification [27].

### 4.5. Effect of Hoagland Solution + Bacillus subtilis L1-21 on HLB Asymptomatic Citrus Plants

HLB asymptomatic citrus plants which were grown under greenhouse of Yunnan Agricultural University, Kunming, China was selected for application of different treatments. Four treatments (50% Hoagland solution, 50% Hoagland solution + *B. subtilis* L1-21, *B. subtilis* L1-21 and ddH_2_O) with three replications were applied to check the control effect on *C*Las titer in citrus plants. Leaf samples were collected before and after 20, 40, and 60 days of treatment. Leaf samples were placed in a cool box with ice and brought to the laboratory for isolation of endophytes. DNA extraction and real-time qPCR analysis was performed as mentioned above. Isolation of endophytes and pathogen abundance were checked through previous method as described above.

### 4.6. Effect of Hoagland Solution + Bacillus subtilis L1-21 on HLB Symptomatic Citrus Plants

HLB-affected citrus plants (*C. lemon*) were selected which were grown under greenhouse of Yunnan Agricultural University, Kunming, China. Four treatments (50% Hoagland solution, 50% Hoagland solution+ *B. subtilis* L1-21, *Bacillus subtilis* L1-21 and ddH_2_O) with three replications were applied to check the control effect on *C*Las titer in citrus plants. Leaf samples were collected before and after 20, 40, and 60 days of treatment. The samples were placed in a cool box with ice and brought to the laboratory for isolation of endophytes. DNA extraction, qPCR for pathogen abundance, and endophytes isolation were evaluated as described above.

### 4.7. Statistical Analysis

All the experiments were conducted with three replicates in each treatment. The data were statistically analyzed using analysis of variances (ANOVA) in IBM SPSS Statistics 23, the means were subjected to Duncan’s multiple range test at *p* ≤ 0.05. All figures were processed and analyzed using Adobe Illustrator CS5 (Adobe Systems Inc., San Francisco, CA, USA) and GraphPad Prism (8.0.2).

## 5. Conclusions

**Figure 4:**
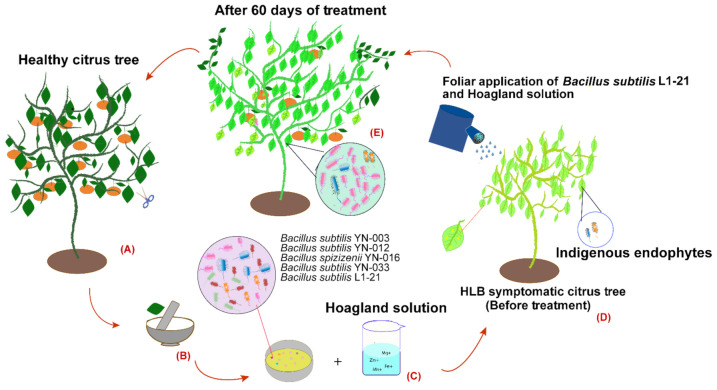
Concluding sketch of experiments performed in this study. (**A**) Healthy citrus tree. (**B**) Isolation of indigenous endophytic bacteria from leaves of healthy citrus tree. (**C**) Colony growth of different endophytic bacteria. (**D**) Foliar application of *Bacillus subtilis* L1-21 with combination of Hoagland solution on HLB-symptomatic citrus tree. (**E**) After 60 days of continuous treatment, citrus tree became healthy with reduce number of *C*Las pathogen.

Here, we describe that endophyte *B. subtilis* L1-21 in the presence of nutrient solution has noticeable effect on pathogen reduction inside diseased citrus plants. However, the complex interactions need to be uncovered in more detail. Series of events performed in this study are mentioned in summarized sketch in Figure 4. Hoagland solution as a nutrient source could change plant structure in asymptomatic plants. In addition, we found that micro-nutrients alone could not make enough difference to the reduction of pathogen, and pathogen numbers were stable. Remarkable consistency of these endophytes present in citrus is due to the time interval of endophytes check. It was also concluded that *C*Las reduction has an inverse relationship with *B. subtilis* L1-21 colonization. Citrus endophytes are suggested as environmentally friendly control strategies, which can potentially strengthen the native microbial diversity of citrus plants and finally overcome pathogen spread.

## Figures and Tables

**Table 1:** Antagonistic effect of selected *Bacillus* isolates against *Xanthomonas citri* subsp. *citri*.

Bacillus Species	Inhibition Diameter (mm)	Colony Diameter (mm)	Growth Inhibition Ratio (%)
*B. subtilis* YN-003	18.66 ± 0.15	11.33 ± 0.15	0.39 ± 0.007 b
*B. subtilis* YN-012	17.66 ± 0.15	11.33 ± 0.15	0.36 ± 0.014 b
*B. spizizenii* YN-016	15.33 ± 0.15	11.33 ± 0.15	0.26 ± 0.015 c
*B. subtilis* YN-024	12.66 ± 0.15	9.66 ± 0.15	0.23 ± 0.019 c
*B. subtilis* YN-033	15.33 ± 0.15	10.33 ± 0.15	0.33 ± 0.014 c
*B. subtilis* L1-21	24.33 ± 0.15	9.00 ± 0.26	0.63 ± 0.009 a

Significance difference (*p* < 0.05) between growth inhibitions ratios (%) of different isolated strains are indicated by different letters according to Duncan’s Multiple Range Test at *p* < 0.05 of three replicates. Growth inhibition ratio = [(Inhibition zone diameter − Colony diameter)/Inhibition zone diameter] × 100.

**Table 2:** Number of pathogen abundance of *C*Las in HLB-affected citrus leaves before and after treatment of candidate *Bacillus* sp. with and without combination of Hoagland solution by using half leaf method.

Treatment	Leaf Samples	Pathogen Abundance/g				
		0 Day	1st Day	2nd Day	3rd Day	4th Day
T1	0–1	8.72 × 10^6^	1.27 × 10^5^			
	0–2	6.47 × 10^5^		1.09 × 10^4^		
	0–3	5.24 × 10^5^			3.34 × 10^5^	
	0–4	9.48 × 10^5^				8.79 × 10^5^
T2	0–1	9.33 × 10^5^	1.80 × 10^5^			
	0–2	4.17 × 10^5^		3.32 × 10^5^		
	0–3	1.34 × 10^5^			1.11 × 10^5^	
	0–4	1.47 × 10^6^				9.11 × 10^5^
T3	0–1	2.65 × 10^5^	4.12 × 10^4^			
	0–2	7.52 × 10^4^		5.75 × 10^4^		
	0–3	3.25 × 10^5^			2.53 × 10^4^	
	0–4	3.11 × 10^5^				5.24 × 10^4^
T4	0–1	8.76 × 10^5^	1.01 × 10^5^			
	0–2	5.71 × 10^5^		3.29 × 10^5^		
	0–3	5.89 × 10^5^			2.66 × 10^5^	
	0–4	5.96 × 10^5^				4.48 × 10^5^
T5	0–1	6.58 × 10^5^	4.34 × 10^5^			
	0–2	8.56 × 10^6^		6.12 × 10^6^		
	0–3	9.82 × 10^6^			5.82 × 10^6^	
	0–4	2.76 × 10^6^				9.33 × 10^5^
T6	0–1	1.03 × 10^6^	8.54 × 10^5^			
	0–2	7.41 × 10^5^		3.09 × 10^5^		
	0–3	1.48 × 10^6^			4.20 × 10^5^	
	0–4	6.21 × 10^4^				4.16 × 10^3^
T7	0–1	5.12 × 10^5^	3.03 × 10^5^			
	0–2	2 × 10^4^		1.55 × 10^4^		
	0–3	2.58 × 10^6^			9.48 × 10^5^	
	0–4	3.01 × 10^5^				1.34 × 10^5^
T8	0–1	2.03 × 10^5^	2.56 × 10^4^			
	0–2	1.31 × 10^6^		1.22 × 10^6^		
	0–3	8.68 × 10^5^			5.72 × 10^5^	
	0–4	9.29 × 10^6^				1.17 × 10^6^
T9	0–1	5.14 × 10^6^	2.86 × 10^5^			
	0–2	2.62 × 10^6^		2.83 × 10^5^		
	03	9.41 × 10^5^			8.58 × 10^4^	
	0–4	6.71 × 10^4^				6.16 × 10^3^
T10	0–1	6.19 × 10^6^	2.63 × 10^6^			
	0–2	1.07 × 10^6^		1.60 × 10^5^		
	0–3	1.65 × 10^6^			8.16 × 10^5^	
	0–4	1.65 × 10^5^				7.52 × 10^3^
T11	0–1	7.78 × 10^6^	5.21 × 10^6^			
	0–2	6.47 × 10^6^		5.19 × 10^6^		
	0–3	8.77 × 10^5^			8.06 × 10^5^	
	0–4	4.66 × 10^6^				4.12 × 10^6^
T12	0–1	3.57 × 10^5^	6.43 × 10^5^			
	0–2	8.43 × 10^5^		8.33 × 10^6^		
	0–3	5.20 × 10^6^			5.05 × 10^6^	
	0–4	5.15 × 10^5^				7.27 × 10^5^

Pathogen abundance in each sample were calculated using standard curve used in our previous study [27]. Every treatment consists of three replications and each replicates consist of 12 citrus leaves used for midribs analysis from which the pathogen abundance was calculated. T1 = *Bacillus subtilis* YN-003, T2 = *B. subtilis* YN-003 + Hoagland solution, T3 = *B. subtilis* YN-012, T4 = *B. subtilis* YN-012 + Hoagland solution, T5 = *B. spizizenii* YN-016, T6 = *B. spizizenii* YN-016 + Hoagland solution, T7 = *B. subtilis* YN-033, T8 = *B. subtilis* YN-033, T9 = *B. subtilis* L1-21, T10 =*B. subtilis* L1-21 + Hoagland solution, T11 = Hoagland solution, T12 = Control(ddH_2_O), 0–1, 0–2, 0–3, 0–4 means leaf samples at 0 Day.

**Table 3:** Pathogen abundance of *C*Las in HLB asymptomatic citrus plants after foliar application of *Bacillus subtilis* L1-21 and Hoagland solution.

Sr	Treatment	0 Day		20 Day		40 Day		60 Day	
		Ct Value	Pathogen Abundance	Ct Value	Pathogen Abundance	Ct Value	Pathogen Abundance	Ct Value	Pathogen Abundance
T1	50% Hoagland solution	30.20 ± 0.003	5.13 × 10^2^	30.38 ± 0.022	4.12 × 10^2^	30.52 ± 0.023	4.12 × 10^2^	31.24 ± 0.025	4.12 × 10^2^
T2	50% HS + *B. subtilis* L1-21	30.64 ± 0.012	3.20 × 10^2^	31.96 ± 0.024	1.42 × 10^2^	33.69 ± 0.003	1.42 × 10^2^	34.588 ± 0.017	2.74 × 10^1^
T3	*B. subtilis* L1-21	30.02 ± 0.013	5.46 × 10^2^	31.57 ± 0.009	1.69 × 10^2^	32.48 ± 0.016	1.69 × 10^2^	33.79 ± 0.008	1.69 × 10^2^
T4	Control(ddH_2_O)	30.69 ± 0.009	2.95 × 10^2^	29.54 ± 0.016	9.57 × 10^2^	28.66 ± 0.004	9.57 × 10^2^	27.73 ± 0.009	9.57 × 10^2^

Ct = Cycle threshold. HS = Hoagland solution. Pathogen abundance in each sample was calculated through standard curve used in our previous study [27]. Every treatment consists of three replications and each replicate consists of 3 citrus plants (3 years old).

**Table 4:** Huanglongbing symptomatic citrus plants after foliar application of *Bacillus subtilis* L1-21 and Hoagland solution.

Sr	Treatment Name	0 Day		20 Day		40 Day		60 Day	
		CT	Pathogen Abundance	CT	Pathogen Abundance	CT	Pathogen Abundance	CT	Pathogen Abundance
T1	50% Hoagland solution	23.90 ± 0.018	4.18 × 10^4^	24.59 ± 0.015	2.94 × 10^4^	24.32 ± 0.014	2.47 × 10^4^	24.38 ± 0.011	2.45 × 10^4^
T2	50% HS + *B. subtilis* L1-21	21.06 ± 0.015	9.51 × 10^5^	22.28 ± 0.007	1.35 × 10^5^	23.04 ± 0.005	8.25 × 10^4^	25.57 ± 0.088	1.06 × 10^4^
T3	*B. subtilis* L1-21	23.13 ± 0.016	8.17 × 10^4^	24.47 ± 0.005	3.04 × 10^4^	24.95 ± 0.002	2.29 × 10^4^	28.12 ± 0.002	2.08 × 10^3^
T4	ddH_2_O (Ck)	22.08 ± 0.012	1.92 × 10^5^	22.25 ± 0.005	1.58 × 10^5^	22.84 ± 0.013	1.08 × 10^5^	22.26 ± 0.007	1.39 × 10^5^

CT = Threshold cycle. HS = Hoagland solution. Pathogen abundance in each sample were calculated using the standard curve used in our previous study [27]. Every treatment consists of three replications and each replicates consist of 3 citrus plants (3 years old).

## Data Availability

All the data is present inside manuscript file.
